# hERG K^+^ Channels Promote Survival of Irradiated Leukemia Cells

**DOI:** 10.3389/fphar.2020.00489

**Published:** 2020-04-24

**Authors:** Daniela Palme, Milan Misovic, Katrin Ganser, Lukas Klumpp, Helmut R. Salih, Daniel Zips, Stephan M. Huber

**Affiliations:** ^1^Department of Radiation Oncology, University Hospital Tübingen, Tübingen, Germany; ^2^Clinical Collaboration Unit Translational Immunology, German Cancer Consortium (DKTK), University Hospital Tübingen, Tübingen, Germany; ^3^German Cancer Consortium (DKTK), Partner Site Tübingen, Tübingen, Germany; ^4^German Cancer Research Center (DKFZ), Heidelberg, Germany

**Keywords:** ionizing radiation, patch-clamp whole-cell recording, flow cytometry, hERG1 potassium channels, S progression, G_2_/M arrest

## Abstract

Many tumor cells express highly elevated activities of voltage-gated K^+^ channels in the plasma membrane which are indispensable for tumor growth. To test for K^+^ channel function during DNA damage response, we subjected human chronic myeloid leukemia (CML) cells to sub-lethal doses of ionizing radiation (0–8 Gy, 6 MV photons) and determined K^+^ channel activity, K^+^ channel-dependent Ca^2+^ signaling, cell cycle progression, DNA repair, and clonogenic survival by whole-cell patch clamp recording, fura-2 Ca^2+^ imaging, Western blotting, flow cytometry, immunofluorescence microscopy, and pre-plating colony formation assay, respectively. As a result, the human erythroid CML cell line K562 and primary human CML cells functionally expressed hERG1. Irradiation stimulated in both cell types an increase in the activity of hERG1 K^+^ channels which became apparent 1–2 h post-irradiation. This increase in K^+^ channel activity was paralleled by an accumulation in S phase of cell cycle followed by a G_2_/M cell cycle arrest as analyzed between 8 and 72 h post-irradiation. Attenuating the K^+^ channel function by applying the hERG1 channel inhibitor E4031 modulated Ca^2+^ signaling, impaired inhibition of the mitosis promoting subunit cdc2, overrode cell cycle arrest, and decreased clonogenic survival of the irradiated cells but did not affect repair of DNA double strand breaks suggesting a critical role of the hERG1 K^+^ channels for the Ca^2+^ signaling and the cell cycle control during DNA damage response.

## Introduction

Tumor cells express ion channel toolkits that differ from that of their healthy parental counterparts. Importantly, this altered ion channel expression serves pivotal functions in neoplastic transformation, survival, proliferation, migration, invasion and metastasis, or therapy resistance of tumor cells suggesting ion channels as potential targets in antitumor therapy. Notably, certain individual types of ion channels are overexpressed in several different tumor entities classifying them as channel with high oncogenic function [for review see (Huber, 2013)]. Among those are ether-à-go-go-related (hERG1, K_v_11.1, *KCNH2*) voltage-gated, fast inactivating human K^+^ channels (Vandenberg et al., 2012) that have been reported in several tumor entities including chronic myeloid leukemia (CML) cells (Arcangeli, 2005). Non-neoplastic bone marrow CD34^+^/CD38^–^ hematopoietic stem cells (Li et al., 2008) and resting or proliferating lymphocytes (Smith et al., 2002), in contrast, do not express hERG1.

hERG1 has been demonstrated predominantly by the seminal work of Annarosa Arcangeli’s group in Florence, Italy, to accomplish outside-in signaling in adhesomes that are macromolecular signaling complexes at focal adhesion sites. hERG1 channels physically interact with receptors in the plasma membrane such as β1-integrin [for review see (Becchetti et al., 2019)]. Functionally, hERG1 may contribute to survival, proliferation (Wang et al., 2002), and metastasis of tumor cells (Manoli et al., 2019). Moreover, hERG1 reportedly may promote tumor vascularization (Crociani et al., 2013) and confer resistance against chemotherapeutics (Pillozzi et al., 2011).

The vast majority of CML carries the Philadelphia chromosome that results from a reciprocal translocation of chromosomes 9 and 22. This translocation gives rise to the Bcr-Abl (breakpoint cluster region/Abelson) fusion oncogene which encodes a constitutively active tyrosine kinase, and tyrosine kinase inhibitors (TKI) such as imatinib are the first line therapy for CML. For a selected patient group (e.g., with TKI resistant tumors), however, hematopoietic stem cell transplantation after myeloablative conditioning remains a curative therapy option. Conditioning may involve total body irradiation and disease relapse is together with transplant toxicity the major cause of transplant failure in patients allografted for CML (Craddock, 2018). Notably, ionizing radiation has been demonstrated to activate K^+^ channels which in turn contribute to DNA damage response and survival of the irradiated tumor cells [for review see (Huber et al., 2013)].

In particular, our previous work has demonstrated K_v_3.4 K^+^ channel activity in CML cells which is stimulated by ionizing radiation and which contributes to DNA damage response and survival of these cells (Palme et al., 2013). Given the upregulation of hERG1 in leukemia (see above), the present study aimed to test for a function of hERG1 channels in the stress response of leukemia cells treated with ionizing radiation. In addition, the crosstalk of K_v_3.4 K^+^ and hERG1 channels in irradiated CML cells should be characterized.

To this end, K562 CML cells and—in further experiments—primary CML cells were used as *in vitro* models since K562 cells reportedly express hERG1 (Smith et al., 2002) and respond to ionizing radiation with elevated K_v_3.4 (Palme et al., 2013) and other plasmalemmal ion channel activity and Ca^2+^ signaling (Heise et al., 2010). The present study applied patch-clamp fast whole cell recording, fura-2 Ca^2+^ imaging, immunoblotting, flow cytometry, immunofluorescence microscopy, and colony formation assay to analyse radiogenic hERG1 activation, hERG1-dependent Ca^2+^ signaling and activation of Ca^2+^ effector proteins, bromodeoxyuridine (BrdU) incorporation and cell cycle progression, repair of DNA double-strand breaks, as well as cell death and clonogenic survival in irradiated CML cells.

## Material and Methods

### Cell Culture

Primary CML cells were isolated by density gradient centrifugation after obtaining informed consent in accordance with the Helsinki protocol, and the study was performed according to the guidelines of the local ethics committee. Primary CML cells and K562 human erythroid CML cells were cultivated in Roswell Park Memorial Institute (RPMI) 1640 medium containing l-glutamine (Gibco, Karlsruhe, Germany) supplemented with 10% fetal calf serum (FCS) and penicillin (100 U/ml)/streptomycin (100 µg/ml). Ionizing radiation (6 MV photons, single dose of 1–8 Gy) was applied by using a linear accelerator (LINAC SL25 Philips) at a dose rate of 4 Gy/min at room temperature. Following irradiation, cells were post-incubated in supplemented RPMI 1640 medium for 1–72 h (immunoblotting, patch-clamp, fura-2 Ca^2+^-imaging, flow cytometry) and 2 weeks (colony formation).

### Blockage of hERG1 and K_v_3.4

According to a meta study (Polak et al., 2009) reported IC_50_ values for the blockage of hERG1 by the class III antiarrhythmic agent E4031 in expression systems *in vitro* range from 8 to 570 nM (mean 81 nM, median 17 nM, n = 14) which suggests a quantitative channel inhibition at a concentration around 200–800 nM in serum-free buffer solution. To compensate for binding to plasma proteins (Webster et al., 2001) and time-dependent drug degradation we applied in initial experiments 3 µM E4031, later on, we reduced to 1 µM. E4031 was initially dissolved in DMSO (< 0.1% DMSO final concentration). Further batches were dissolved in _dd_H20. E4031-DMSO control, vehicle (DMSO), was added at the same concentration. To the best of our knowledge, E4031 at the applied concentration does not interfere with the non-hERG1 channels detected in K562 cells. Tetraethylammonium (TEA) which was used at a concentration of 3 mM to inhibit K_v_3.4 channels does not exert relevant blockage of hERG1 channels [hERG1 IC_50_ = 50 mM TEA (Choi et al., 2011)]. For 3 mM TEA-containing NaCl solution (see below), 3 mM NaCl was replaced isosmotically by diluting 150 mM TEA solution with NaCl solution (see below) by a factor of 1:50.

### Patch-Clamp Recording

**Figure 1:**
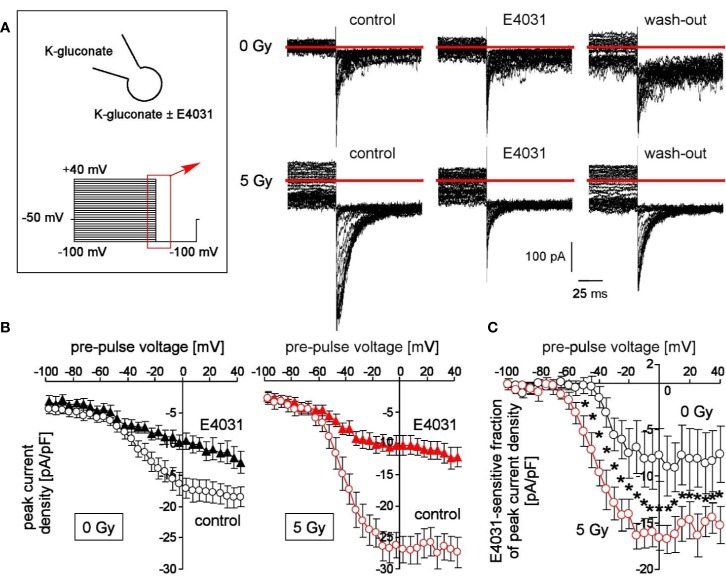
Irradiation modifies voltage-dependent hERG channels in K562 cells. **(A)** Current tracings recorded from a control (0 Gy, upper line) and a 5 Gy-irradiated cell (lower line). Records were obtained with K-d-gluconate pipette and bath solution during a hyperpolarizing voltage step to −100 mV delivered from pre-pulses ranging from −100 to +100 mV (5 mV increments, see insert). Deactivating inward tail currents were recorded before (control), during (E4031) and after (wash-out) of the hERG1 inhibitor E4031 (1 µM). Red line indicates zero current. **(B)** Relationships between mean (± SE, n = 9–16) peak current density of the deactivating tail currents at −100 mV clamp voltage and the pre-pulse voltage in control (0 Gy, left) and 5 Gy-irradiated (right) K562 cells recorded as in **(A)** before (open circles) and during administration of E4031 (closed triangles). **(C)** Relationships between mean (± SE, n = 6–9) E4031-sensitive fractions of peak current density and activating pre-pulses in control (0 Gy, black circles) and 5 Gy-irradiated (red circles) as recorded in **(A)**. * indicates p ≤ 0.05, two-tailed (Welch-corrected) t-test.

K562 and primary CML cells were irradiated with 0 or 5 Gy. 1–4 h post irradiation, fast hERG1-mediated deactivating whole-cell tail currents were evoked by voltage square pulses delivered from different holding potentials/pre-pulses to voltages of −80 mV or −100 mV as indicated in the inserts of **Figures 1A**, **6A**. Currents were recorded (10 kHz sampling rate) and 3-kHz low-pass-filtered by an EPC-9 amplifier (HEKA, Lambrecht, Germany) using Pulse software (HEKA) and an ITC-16 Interface (InstruTech, Port Washington, NY, USA). Borosilicate glass pipettes (~5 MΩ pipette resistance; GC150 TF-10, Clark Medical Instruments, Pangbourne, UK) manufactured by a microprocessor-driven DMZ puller (Zeitz, Augsburg, Germany) were used in combination with a scanning tunneling microscope (STM) electrical micromanipulator (Lang GmbH and Co KG, Germany).

Cells were continuously superfused at 37°C temperature with NaCl solution [in mM: 125 NaCl, 32 N-2-hydroxyethylpiperazine-N-2-ethanesulfonic acid (HEPES), 5 KCl, 5 d-glucose, 1 MgCl_2_, 1 CaCl_2_, 0 or 0.001 E4031, titrated with NaOH to pH 7.4]. Superfusion (~1 ml/min) was applied through a heated flow system inserted into the dish (bath volume approximately 200 µl). The bath was grounded *via* a bridge filled with NaCl bath solution). Upon GΩ-seal formation (10–100 GΩ seal resistance) and entry into the whole-cell recording mode, cells were recorded with K-d-gluconate bath solution (in mM: 150 K-d-gluconate, 10 HEPES, 1 Ca-d-gluconate_2_, titrated with KOH to pH 7.4) and K-d-gluconate pipette solution (in mM: 140 K-d-gluconate, 5 HEPES, 5 MgCl_2_, 1 K_2_-ethylene glycol-bis(-aminoethyl ether)-N,N,N′,N′-tetraacetic acid (EGTA), 1 K_2_-ATP, titrated with KOH to pH 7.4).

Clamp voltages refer to the cytosolic face of the plasma membrane and were corrected offline for the applying liquid junction potential according to Barry and Lynch (Barry and Lynch, 1991). In particular, for calculation of the liquid junction potentials relative mobilities of 1.0388, 0.33, 0.3, 1.0, 0.682, 0.488, and 0.361 were assumed for chloride, gluconate, HEPES, potassium, calcium, sodium, and magnesium, respectively. The set-off liquid junction potential (as measured against the earthed bridge connected to the bath both containing NaCl solution) between K-d-gluconate pipette- and NaCl bath solution was +14.9 mV which applies with −14.9 mV at the intracellular plasma membrane face upon giga seal formation and entry into the whole-cell mode. The liquid junction potential between K-d-gluconate bath solution and NaCl bridge applying at the extracellular membrane face upon bath exchange was calculated to −14.6 mV suggesting an effective deviation from the adjusted clamp voltage of −0.3 mV. Inward currents are defined as influx of cations into the cells (or efflux of anions out of the cell), depicted as downward deflections of the current tracings, and defined as negative currents in the current voltage relationships. Peak currents were analyzed.

### Fura-2 Ca^2+^ Imaging

Fluorescence measurements were performed by the use of an inverted phase-contrast microscope (Axiovert 100; Zeiss, Oberkochen, Germany). Fluorescence was evoked by a filter wheel (Visitron Systems, Puchheim, Germany)-mediated alternative excitation at 340/26 or 387/11 nm (AHF, Analysentechnik, Tübingen, Germany). Excitation and emission light were deflected by a dichroic mirror (409/LP nm beamsplitter, AHF) into the objective (Fluar x40/1.30 oil; Zeiss) and transmitted to the camera (Visitron Systems), respectively. Emitted fluorescence intensity was recorded at 587/35 nm (AHF). Excitation was controlled and data acquired by Metafluor computer software (Universal Imaging, Downingtown, PA, USA). The 340/380-nm fluorescence ratio was used as a measure of cytosolic free Ca^2+^ concentration (c[Ca^2+^]_free_). K562 cells were irradiated (0 or 5 Gy) and loaded with fura-2/AM (2 µM for 30 min at 37°C; Molecular Probes, Göttingen, Germany) in supplemented RPMI medium. Steady state c[Ca^2+^]_free_ was recorded in irradiated (0 or 5 Gy) K562 cells (1–4 h post-irradiation) in the continuous presence of external Ca^2+^ during superfusion with Ca^2+^-containing NaCl solution (see above) before, during, and after administration of E4013 (1µM) or TEA (3 mM).

### Western Blotting

Irradiated K562 cells (0 and 5 Gy, 2 h post-radiation) were lysed in a buffer (containing in mM: 50 HEPES, pH 7.5, 150 NaCl, 1 ethylenediaminetetraacetic acid (EDTA), 10 sodium pyrophosphate, 10 NaF, 2 Na_3_VO_4_, 1 phenylmethylsulfonylfluorid (PMSF) additionally containing 1% Triton X-100, 5 µg/ml aprotinin, 5 µg/ml leupeptin, and 3 µg/ml pepstatin) and separated by sodium dodecyl sulfate-polyacrylamide gel electrophoresis (SDS-PAGE) under reducing condition. In some experiments cells were pre-incubated (0.5 h), irradiated and post-incubated (2 h) in the presence of the hERG1 channel inhibitor E4013 (0 or 1 µM). SDS-PAGE-segregated proteins were electro-transferred onto PVDF membranes (Roth, Karlsruhe, Germany). Blots were blocked in Tris-buffered saline (TBS) buffer containing 0.05% Tween 20 and 5% non-fat dry milk for 1 h at room temperature. The membrane was incubated overnight at 4°C with the following primary antibodies (in TBS-Tween / 5% milk): rabbit anti-phospho-CaMKII (Thr286) antibody (Cell Signaling #3361, New England Biolabs, Frankfurt, Germany, 1:1,000), rabbit anti-CaMKII (pan) antibody (Cell Signaling #3362, 1:1,000), rabbit anti-phospho-cdc2 (Tyr15) (Cell Signaling #9111, 1:1,000), or β-actin (mouse anti-β-actin antibody, clone AC-74, Sigma #A2228 1:20,000). Antibody binding was detected with a horseradish peroxidase-linked goat anti-rabbit IgG antibody or anti-mouse IgG antibody (Cell Signaling # 7074 and #7076, respectively; 1:1,000–1:2,000 dilution in TBS-Tween/5% milk) incubated for 1 h at room temperature and enhanced chemiluminescence (ECL Western blotting analysis system, GE Healthcare/Amersham-Biosciences, Freiburg, Germany).

### Bromodeoxyuridine Incorporation

To test for DNA synthesis/repair by bromodeoxyuridine (BrdU) incorporation, the cells were resuspended in supplemented RPMI 1640 medium containing BrdU (20 µM) and E4031 (0 or 3 µM) irradiated with 0 or 5 Gy, post-incubated for 8 h at 37°C, washed, and fixed with 70% ethanol and consecutively treated with RNase A (0.1 mg/ml in PBS for 10 min at 37°C), pepsin (0.5 mg/ml in 0.05 N HCl for 10 min at 37°C), and 2 N HCl (for 10 min at room temperature). For immunolabelling, cells were incubated (30 min at room temperature) with a monoclonal mouse anti-BrdU antibody [1:67 dilution in phosphate buffered saline (PBS)/1% bovine serum albumin (BSA), Becton Dickinson, Pharmingen, Freiburg, Germany] and post-incubated (30 min at room temperature) with a fluorescein isothiocyanate (FITC)-conjugated rabbit anti-mouse IgG antibody (1:100 in PBS/1% BSA, Dako, Hamburg, Germany). Thereafter, cells were stained (15 min at 4°C) with propidium iodide solution (25 µg/ml propidium iodide and 20 µg/ml RNAse A in PBS/1% BSA). BrdU- and propidium iodide-specific fluorescence were analyzed by flow cytometry (FACSCalibur, Becton Dickinson, Heidelberg, Germany, 488 nm excitation wavelength) in fluorescence channels FL-1 (515–545 nm emission wavelength) and FL-3(> 670 nm emission wavelength), respectively. Data were analyzed with the FCS Express 3 software (De Novo Software, Los Angeles, CA, USA).

### Cell Cycle Analysis in Flow Cytometry

K562 or primary CML cells were pre-incubated (30 min), irradiated (0 or 5 Gy), and incubated for further 24–72 h in supplemented RPMI 1640 medium additionally containing E4031 (0, 1, or 3 µM). For cell cycle analysis, cells were permeabilized and stained (30 min at room temperature) with Nicoletti propidium iodide solution (containing 0.1% Na-citrate, 0.1% Triton X-100, 10 µg/ml propidium iodide in PBS), and the DNA amount was analyzed by flow cytometry in fluorescence channel FL-3 (linear scale). In parallel, cells with degraded DNA were defined by the subG_1_ population of the propidium iodide histogram recorded in fluorescence channel FL-2 (logarithmic scale, 564–606 nm emission wavelength).

### Colony Formation Assay

To test for clonogenic survival, K562 cells were plated in six-well plates (n = 100 cells/well) in supplemented RPMI 1640 medium further containing E4031 (0 or 1 µM), irradiated (0, 2, 4, 6 Gy), and post-incubated for 2 weeks. Thereafter, colonies were defined as cluster of ≥ 50 cells and colony number N counted. Plating efficiency PE was defined by ratio between counted colonies and plated cells (PE = N/n). The survival fraction (SF) was calculated by normalizing in both arms (E4031 and vehicle control) separately the plating efficiency after irradiation (PE_xGy_) to that of the corresponding unirradiated control (PE_0Gy_) by the formula SF = PE_xGy_/PE_0Gy_.

### γH2AX-Foci Formation

K562 cells were placed in cell culture medium containing E4031 (0 or 3 µM), irradiated (0 or 4 Gy), and post-incubated for 24 h. After several washing steps (PBS), cells were transferred on object slides by cytospin, fixed in 4% formaldehyde in PBS (3 x 5 min at room temperature), and cell membranes were solubilized with 0.1% Triton X-100 in PBS (3 x 5 min at room temperature), washed (PBS), quenched, blocked, incubated with anti-γH_2_AX antibody (Upstate, Millipore, Billerica, MA, clone JBW301; 1:1,000 at room temperature for 1 h), as well as antibody binding visualized by fluorescence microscopy by the use of Alexa Fluor 488 Tyramide Super Boost Kit goat anti-mouse IgG (#B40912, Thermo Fisher Scientific, Schwerte, Germany) according to the protocols supplied by the manufacturer. After additional washing steps (PBS) cells were incubated with 4′,6-diamidino-2-phenylindole (DAPI) (Sigma Aldrich D9542, 25 µg/ml in PBS, 10 min at room temperature, dark conditions). Object slides were mounted in Fluorescence Mounting Medium (Dako, Carpinteria, CA) and z-stack fluorescence images (slice thickness = 0.5 µm, z-stacks = 11) were acquired using ApoTome.2 (Zeiss, Oberkochen, Germany). Nuclear γH2AX-foci were then counted in Maximum Intensity Projection images generated from the z-stacks.

### Statistics

Data are given as means ± SE or individual values plus median, statistical significance was estimated by (Welch-corrected) two-tailed and one-sample t-test or for multiple pair comparisons by ANOVA. An error probability of p ≤ 0.05 was considered statistically significant.

### Safety Guidelines

All experimental protocols and research activities adhered to the standard biosecurity and institutional safety guidelines.

## Results

To test for radiation-stimulated hERG1 function in K562 cells, fast whole-cell currents of irradiated (0 or 5 Gy, 1–4 h post-irradiation) were recorded with the patch-clamp technique using K^+^ and d-gluconate^−^ as principle charge carriers in both pipette and bath solution. Since permeability of the plasma membrane for the large anion d-gluconate^−^ is usually low, this configuration measures primarily K^+^-selective (or nonselective cation) currents. hERG1 currents stand out due to their unusual gating characteristics: hERG1 channels activate slowly and inactivate rapidly during depolarizing voltage steps. Upon repolarization of the membrane voltage, release from inactivation occurs with faster kinetics than deactivation of the channels giving rise to deactivating tail currents (Vandenberg et al., 2012).

Here, we applied voltage pre-pulses delivered from −50 mV holding potential to voltages between −100 and +40 mV, followed by a hyperpolarizing voltage step to −100 mV (**Figure 1A**, insert). This hyperpolarization of the plasma membrane elicited deactivating tail inward currents (**Figure 1A**). Importantly, these tail currents were larger in pre-irradiated (5 Gy) than in control (0 Gy) cells (compare upper and lower line in **Figure 1A**). Moreover, bath administration of E4031 (1 µM) inhibited in a more or less reversible manner a fraction of the tail currents (**Figure 1A**, lower line) suggesting an involvement of hERG1 channels. **Figure 1B** shows the mean peak current densities at −100 mV voltage in dependence on the pre-pulse voltage of control (0 Gy, left) and irradiated (5 Gy, right) K562 cells recorded before (open circles) and during hERG1 inhibition (closed triangles). Irradiation significantly increased the E4031-sensitve fraction of deactivating tail current densities (compare black and red symbols in **Figure 1C**). Notably, ionizing radiation did not alter whole-cell capacities *c* as a measure membrane area (*c*_0Gy_ = 11.5 ± 0.5 pF *vs.* *c*_5Gy_ = 11.0 ± 0.8 pF, n = 9–16) suggesting that the increased hERG1 current did not just result from a radiation-induced increase in cell volume.

**Figure 2:**
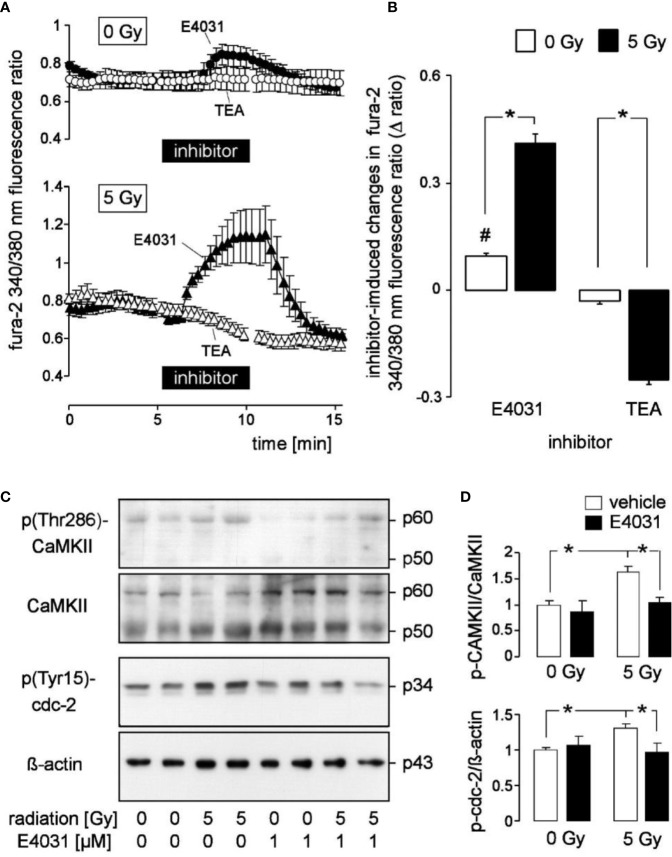
Radiation augments in K562 cells the modulation of cytosolic free Ca^2+^ concentration (c[Ca^2+^]_free_) by the hERG1 inhibitor E4031 and the K_v_3.4 blocker tetraethylammonium (TEA): effect of E4031 on Ca^2+^-regulated signaling. **(A)** Time course of the mean (± SE, n = 7–10) ratio between the 340 nm- and the 380 nm-excited fura-2 fluorescence as a measure of c[Ca^2+^]_free_. Ratios were recorded 2–3 h post-irradiation with 0 Gy (upper line, n = 27) and 5 Gy-irradiated (lower line) K562 cells before, during, and after administration of E4031 (1 µM, closed triangles) or TEA (open circles). **(B)** Mean (± SE) change in fura-2 340/380 nm ratio (Δ ratio) elicited in 0 Gy (open bars) and 5 Gy (closed bars)-irradiated cells by E4031 (n = 90–111, left) and TEA (n = 57–59, right) application as recorded in **(A)**. **(C)** Immunoblots showing the abundance of phosphorylated (Thr286, first panel) and total CaMKII isoforms (second panel), phospho-(Tyr15)-cdc2 (third panel), or ß-actin (fourth panel) as loading control in total lysates of K562 cells cropped 2 h after irradiation with 0 and 5 Gy. Cells were irradiated and post-incubated in the absence or presence of the hERG-inhibitor E4031 (1 µM). **(D)** Mean (± SE, n = 3) normalized ratio between phospho-(Thr286)-CaMKII and total CaMKII protein abundance (top) and between phospho-(Tyr15)-cdc2 and ß-actin protein [densitometrically semi-quantified data from **(C)**]. ^#^ indicates different from 0, p ≤ 0.05, one-sample t-test (**B**, 1^st^ column); * indicates p ≤ 0.05, two-tailed (Welch-corrected) t-test in **(B)** and ANOVA in **(D)**.

Radiogenic K^+^ channel activity may modulate Ca^2+^ signaling. In the present study, we did not observe under control conditions gross differences in steady state fura-2 340/380 nm fluorescence ratio as a measure of c[Ca^2+^]_free_ between unirradiated and 5 Gy-irradiated K562 cells when recorded in the continuous presence of external Ca^2+^ (compare the initial parts of the graphs in **Figure 2A** between 0 Gy- and 5 Gy-irradiated cells). The low spatial/temporal resolution of our Ca^2+^ imaging set-up, however, was not suited to detect, e.g., local Ca^2+^ sparks or Ca^2+^ oscillations with frequencies faster than 0.1 Hz. Notably, E4031 evoked in unirradiated K562 cells a faint but significant increase in steady state c[Ca^2+^]_free_ (**Figure 2A**, upper line, closed symbols, and **Figure 2B**, 1^st^ bar) while TEA did not alter steady state c[Ca^2+^]_free_ (**Figure 2A**, upper line, open symbols, and **Figure 2B**, 3^rd^ bar). In irradiated cells, E4031 elicited a much stronger increase in c[Ca^2+^]_free_ than in unirradiated cells and TEA evoked a significant decrease of steady state c[Ca^2+^]_free_ (**Figure 2A**, lower line, **Figure 2B**, 2^nd^ and 4^th^ bars).

The latter observation might result from a Ca^2+^ leak of the plasma membrane that is stimulated in irradiated K562 cells by K_v_3.4. As matter of fact, ionizing radiation has been demonstrated in our previous study (Palme et al., 2013) to accelerate Ca^2+^ re-entry in irradiated Ca^2+^-depleted K562 cells in a K_v_3.4-dependent manner: the K_v_3.4 channel blocker TEA inhibited radiation-stimulated Ca^2+^ re-entry (Palme et al., 2013). To analyze the effect of hERG1 and combined hERG1 and K_v_3.4 blockage on accelerate Ca^2+^ re-entry in irradiated Ca^2+^-depleted K562 cells we applied in the present study a Ca^2+^ removal/re-addition protocol during fura-2 Ca^2+^ imaging (**Supplementary Figure 1A**). As a result, ionizing radiation (5 Gy) stimulated an increase of Ca^2+^ re-entry in Ca^2+^-depleted cells from *s _0Gy-control_* = 0.4 ± 0.03 min^−1^ to *s _5Gy-control_* = 1.0 ± 0.04 min^−1^ (data are means ± SE, n = 167–267, and given as slope *s* of the increase in the fura-2 340/380 nm fluorescence ratio, **Supplementary Figure 1B**). E4031 when administered during Ca^2+^ re-entry augmented the basal (*s _0Gy-E4031_* = 0.9 ± 0.05 min^−1^) and radiation-stimulated (*s _5Gy-E4031_ s* = 2.1 ± 0.04 min^−1^) slope of Ca^2+^ re-entry. Co-administration of E4031 and the K_v_3.4 inhibitor TEA during the Ca^2+^ re-entry, in sharp contrast, re-decreased slope of basal (*s _0Gy-E4031+TEA_* = 0.4 ± 0.04 min^−1^) and radiation-induced (*s _5Gy-E4031+TEA_* = 0.7 ± 0.07 min^−1^) Ca^2+^ re-entry to values observed in the control situations (data not shown) suggesting that K_v_3.4 and hERG1 channels exert opposing effects on Ca^2+^ re-entry in Ca^2+^-depleted cells. This is also illustrated by the calculated radiation-induced fraction of Ca^2+^ re-entry under control situation, in the presence of E4031, and in the presence of E4031 and TEA as shown in **Supplementary Figure 1C**. Combined, the Ca^2+^ removal/re-addition and the Ca^2+^ steady-state experiments of the present study suggest [also when taking into account our previously published data (Palme et al., 2013)] that hERG1 as well as K_v_3.4 contribute to Ca^2+^ signaling in irradiated K562 cells. The opposing effects of hERG and K_v_3.4 inhibition on Ca^2+^ re-entry and steady-state c[Ca^2+^]_free_ further suggest different functions of both channels in the Ca^2+^ signalosome of K562 cells.

Next, we analyzed by immunoblotting potential hERG-stimulated signaling pathways in K562 cells by analyzing the activities of the Ca^2+^ effector proteins CaMKIIs (Ca^2+^/calmodulin-dependent protein kinase II isoforms) and their downstream target cdc2 (cyclin-dependent kinase 1, CDK1) (**Figures 2C, D**). Irradiation (5 Gy) stimulated activation of CaMKIIs and inactivation of their downstream target, cdc2 (cyclin-dependent kinase 1, CDK1) in K562. This was evident from phosphorylation at threonine 286 (CaMKIIs) and tyrosine 15 (cdc2), respectively (1^st^–4^th^ lane in the immunoblots of **Figure 2C** and semi-quantified data shown as open bars in **Figure 2D**). Importantly, hERG1 inhibition by E4031 (1 µM) prevented radiation-induced CaMKIIs activation and cdc2 inactivation (**Figure 2C**, 5^th^–8^th^ lane and **Figure 2D**, closed bars) suggesting that Ca^2+^ signals modulated by hERG1 were required for radiation-induced CaMKIIs activation and inhibition of cdc2.

**Figure 3:**
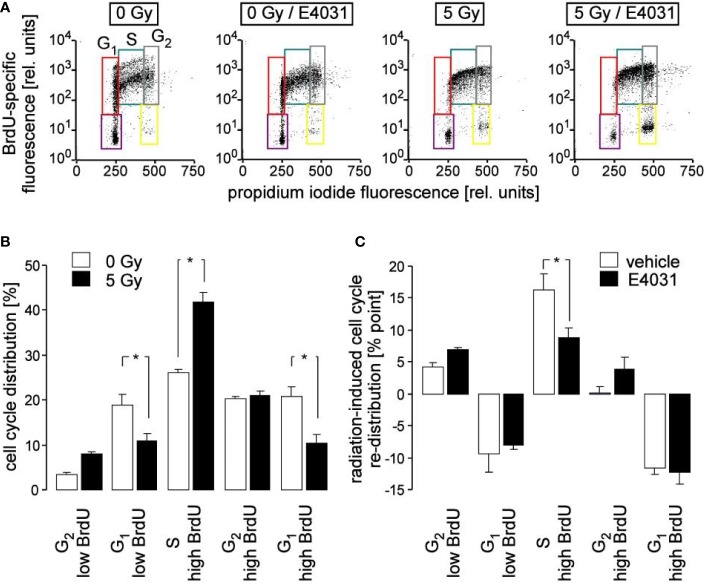
Inhibition of hERG channels stimulates release from S phase arrest. **(A)** Flow cytometry dot plots showing DNA incorporation of the base analogon bromodeoxyuridine (BrdU) and DNA amount by propidium iodide staining during 8 h of incubation following irradiation with 0 Gy (1^st^ and 2^nd^ plot) and 5 Gy (3^rd^ and 4^th^ plot). Irradiation (0 and 5 Gy) and post-incubation (8 h) were carried out either in the absence (1^st^ and 3^rd^ plot) or presence (2^nd^ and 4^th^ plot) of the hERG channel inhibitor E4031 (3 µM). Cells which did not accumulate BrdU and which resided in G_1_ and G_2_ phase of cell cycle are outlined in violet and yellow, respectively. BrdU-positive cells which resided in G_1_, S and G_2_ phase of cell cycle are outlined in red, turquoise, and gray, respectively. **(B, C)** Mean (± SE, n = 8–9) normalized percentage **(B)** and radiation (5 Gy)-induced cell cycle re-distribution (mean percent points ± SE, n = 8–9) of K562 cells **(C)** incubated in the absence (open bars, vehicle) and presence of E4031 (closed bars) residing in the G_2_ low BrdU, G_1_ low BrdU, S, G_2_ high BrdU, or G_1_ high BrdU population as defined in **(A)**. * indicates p ≤ 0.05, ANOVA.

Ca^2+^ signals *via* Ca^2+^ effector proteins such as the multifunctional CaMKI and -II isoforms reportedly control cell cycle progression (Skelding et al., 2011). Therefore, cell cycle progression was analyzed in K562 cells by flow cytometry in dependence on irradiation and hERG1 inhibition. To this end, cells were irradiated with 0 or 5 Gy and post-incubated for 8 h in the presence of bromodeoxyuridine (BrdU) and E4031 (0 or 3 µM). Thereafter, cellular BrdU incorporation and DNA content were visualized by immunofluorescence- and propidium iodide staining, respectively, in flow cytometry experiments (**Figure 3A**). As a result, radiation decreased both G_1_ populations (without and with incorporated BrdU) and increased the BrdU-incorporating S population, increased in tendency BrdU-negative G_2_ population, but left BrdU-positive G_2_ population unchanged (compare open and closed bars in **Figure 3B**). This might be explained by a slowed-down S progression paired with a beginning accumulation of the irradiated cells at the G_2_/M border of cell cycle, or a combination of both. Remarkably, hERG1 inhibition did not modify the radiation-induced decrease of both G1 populations. Instead, E4031 decreased the radiation induced accumulation of BrdU incorporating S phase cells and showed a trend to increase both G_2_ populations (**Figure 3C**), suggestive of a hERG1 function in the radiation-induced S phase arrest during the first 8 h after irradiation.

**Figure 4:**
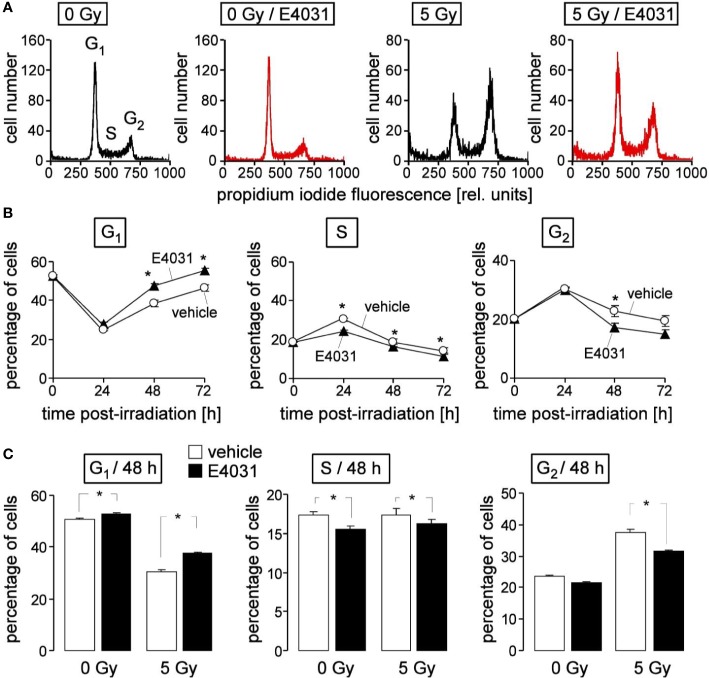
hERG channels delay recovery from radiation-induced release from S and G_2_/M arrest in K562 cells. **(A)** Flow cytometry histograms depicting the propidium iodide fluorescence (Nicoletti staining) of K562 cells irradiated (0 or 5 Gy) and post-incubated (48 h) with vehicle (black) or hERG inhibitor E4031 (3 µM). **(B)** Time course of radiation (5Gy)-induced changes in cell cycle progression in the absence (vehicle, open circles) and presence of E4031 (3 µM, closed triangles). Shown are mean (± SE, n = 21) percentages of cells residing in G_1_ (left), S (middle), or G_2_ (right) phase of cell cycle 0, 24, 48, and 72 h after irradiation. **(C)** Mean percentage (± SE, n = 27) of irradiated (0 or 5 Gy) K562 cells residing 48 h after irradiation in G_1_ (left), S (middle), or G_2_ (right) phase of cell cycle. Cells were pre- and post-incubated with vehicle (open bars) or E4031 (3 µM, closed bars). * indicates p ≤ 0.05, two-tailed t-test in **(B)** and ANOVA in **(C)**.

Next, we studied in K562 cells by flow cytometry using propidium iodine single staining (**Figure 4A**) a potential hERG1 function in cell cycle progression at later time points (24–72 h) after irradiation (0 or 5 Gy). The dynamics of the irradiated cells residing in G_1_, S, or G_2_ phases of cell cycle (**Figure 4B**, open circles) suggest that the proposed initial delay in S phase progression seen at 8 h (see **Figure 3B**) and at 24 h after irradiation transitioned into a G_2_/M arrest (**Figure 4B**, open circles). Later on, the radiation induced decrease of the G_1_ population and the increases in S and G_2_ population regressed again, reaching (almost) basal levels 72 h after irradiation (**Figure 4B**, open circles). hERG1 inhibition with E4031 (3 µM) blunted in the irradiated K562 cells the S phase arrest at 24 h, and accelerated the recovery of G_1_ population and release from G_2_/M arrest between 24 and 72 h after irradiation (**Figure 4B**, closed triangles). In unirradiated K562 cells, E4031 (3 µM for 48 h) slightly increased G_1_ and decreased S population (**Figure 4C**, 1^st^ and 2^nd^ bars) with lower (G_1_) or similar effect size as compared to its action in irradiated cells (**Figure 4C**, 3^rd^ and 4^th^ bars). Combined, the data of **Figures 3** and **4** suggest an involvement of hERG1 in the regulation of cell cycle progression. In particular, in irradiated K562 cells, hERG1 inhibition blunted S and G_2_/M cell cycle arrest.

**Figure 5:**
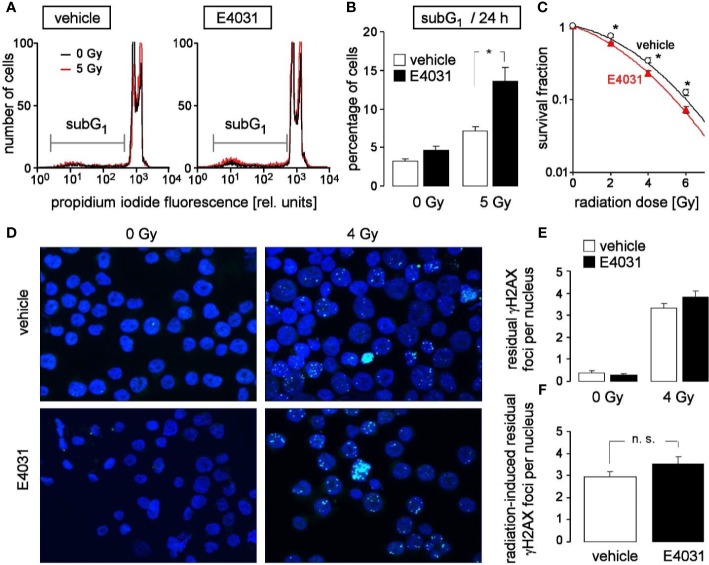
hERG channels confer radioresistance to K562 cells without promoting DNA repair. **(A)** Flow cytometry histograms highlighting the dead population of irradiated (0 Gy in black and 5 Gy in red) K562 cells with low (subG1) propidium iodide fluorescence (Nicoletti staining). K562 cells were irradiated (0 or 5 Gy) and post-incubated (24 h) in the absence (vehicle, left) or presence of hERG inhibitor E4031 (1 µM, right). **(B)** Mean (± SE, n = 9) percentage of dead (subG_1_) K562 cells 24 h after irradiation with 0 (left) or 5 Gy (right) and co-incubation with vehicle alone (open bars) or E4031 (1 µM, closed bars). **(C)** Mean (± SE, n = 12–24) survival fraction of irradiated (0, 2, 4, 6 Gy) K562 cells as determined by pre-plating colony formation assay. Cells were irradiated and post-incubated in the absence (vehicle, black) or presence of E4031 (1 µM, red). **(D)** Fluorescence micrographs of 0 Gy- (left) and 4 Gy-irradiated (closed symbols) K562 cells immunostained against γH2AX (green) and counterstained against DNA with 4′,6-diamidino-2-phenylindole (DAPI) (blue). Cells were irradiated and 24 h post-incubated in the absence or presence of E4031 (3 µM) before analysis. **(E, F)** Mean (± SE, n = 193–273) number of residual nuclear γH2AX foci 24 h after irradiation with 0 (left) or 4 Gy (right) and co-treatment with 0 (open bars) or 3 µM E4031 (closed bars) **(E, F)** of the radiation-induced residual nuclear γH2AX foci 24 h after co-treatment with 0 (open bars) or 3 µM E4031 (closed bars).* and n.s. indicate p ≤ 0.05 and not significant different, respectively, ANOVA in **(B)** and two-tailed (Welch-corrected) t-test in **(C, F)**.

Entry in mitosis with unfixed DNA double strand breaks may result in chromosome aberration and eventually in mitotic catastrophe. To avoid such immature mitosis entry, irradiated cells arrest their cell cycle to gain time for DNA double strand repair. Upon hERG1 inhibition, the observed accelerated release from S and G_2_/M cell cycle arrest might, therefore, impair the survival of irradiated K562 cells. To test for such a scenario, the population of K562 cells with degraded DNA (subG_1_ population) was determined in dependence on hERG1 inhibition (0 or 1 µM E4031) by flow cytometry (propidium iodide Nicoletti staining) 24 h after irradiation with 0 or 5 Gy (**Figure 5A**). As a result, E4031 almost doubled the percentage of dead irradiated cells while having no effect on unirradiated K562 cells (**Figure 5B**). Furthermore, clonogenic survival of irradiated (0, 2, 4, or 6 Gy) K562 cells was determined again in dependence on hERG1 channel inhibition in pre-plating colony formation assay. For this assay, K562 cells were irradiated (0–6 Gy), and thereafter incubated in the absence or presence of E4031 (1 µM) until formation of colonies. For calculation of survival fractions, plating efficiencies (i.e., number of colonies divided by number of plated cells) were normalized to that of the respective 0 Gy control value separately in the E4031 and vehicle treatment arm. The survival fractions were plotted against the radiation dose demonstrating that E4031 decreased survival fractions and, hence, radiosensitized the K562 cells (**Figure 5C**). To test, whether this E4031-mediated radiosensitization is associated with impaired DNA repair, γH2AX foci [phospho-(139S)-H2A histone family member X] were determined as a surrogate marker of residual (= un-repaired) DNA double strand breaks (DSBs) by immunofluorescence microscopy 24 h after irradiation with 0 or 4 Gy (**Figure 5D**). E4031 (3 µM) did not increase the number of residual γH2AX foci (**Figures 5E, F**) suggesting that hERG signaling is not involved in DNA DSB repair. Combined, the data indicate that hERG1 channels contribute to the stress response and clonogenic survival of irradiated K562 cells but do not promote DNA repair.

Mitochondria have been identified to contribute to radiation-induced cell damage by formation of superoxide anion radicals [for review see (Eckert et al., 2019)]. To define a potential role of hERG1 and K_v_3.4 channels herein modulation of inner mitochondrial membrane potential (ΔΨ_m_) and mitochondrial formation of superoxide anion radicals were determined by flow cytometry 24 h after irradiation with 0 or 8 Gy (**Supplementary Figures 2** and **3**). Irradiation and 24 h post-incubation were carried out in the absence or presence of E4031 or TEA, or both inhibitors. As a result, applying the combination of both drugs pronounced hyperpolarization of ΔΨ_m_ and formation of superoxide anions in the irradiated cells. For the latter an additive effect of E4031 and TEA was observed. Together, these data confirmed the contribution of both channels in the stress response of irradiated K562 cells.

**Figure 6:**
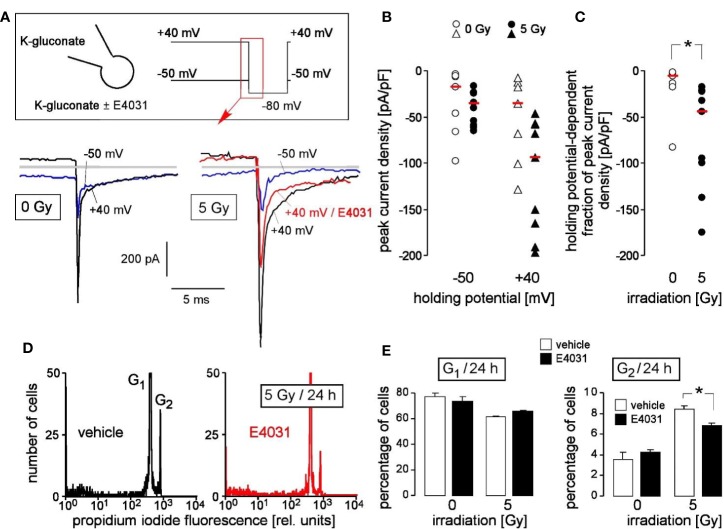
Primary chronic myeloid leukemia (primary CML) cells express radiation-induced hERG currents: involvement in G_2_/M cell cycle arrest. **(A)** Whole-cell deactivating tail current tracings from a control (0 Gy, left) and an irradiated primary CML cell (5 Gy, right). Records were obtained with K-D-gluconate pipette and Na-D-gluconate bath solution (insert). Square pulses to −80 mV (insert) were either delivered from a holding potential of +40 mV (black tracings) or −50 mV (blue tracings) as indicated. The current tracing in red color was recorded during administration of E4031 (3 µM). Gray lines indicate zero current. **(B, C)** Peak current densities of the deactivating tail current after stepping the clamp voltage to −80 mV either from a holding potential of −50 or +40 mV **(B)** and holding potential-dependent peak current density fractions of the deactivating tail current **(C)** of control (0 Gy, open symbols) or 5 Gy-irradiated primary (closed symbols) CML cells. Symbols and red lines indicate individual values (n = 7–9) and medians, respectively. **(D)** Flow cytometry histograms showing the propidium iodide fluorescence (Nicoletti staining) of primary CML cells that were irradiated (5 Gy) and post-incubated (24 h) with vehicle (left) or hERG inhibitor E4031 (3 µM, right). **(E)** Mean (± SE, n = 3–4) percentage of irradiated (0 or 5 Gy) primary CML cells residing 24 h after irradiation in G_1_ (left) or G_2_ (right) phase of cell cycle (right). Cells were pre- and post-incubated with vehicle (open bars) or E4031 (3 µM, closed bars). * indicates p ≤ 0.05, two-tailed (Welch-corrected) t-test in **(C)** and ANOVA in **(E)**.

Finally, primary chronic myeloid leukemia (CML) cells were tested for hERG1 function in the irradiation-induced stress response. Fast whole cell patch clamp recording revealed E4031-sensitive, time-dependently deactivating inward tail currents at −80 mV clamp voltage that exhibited the hERG1-typical dependence on holding potential (**Figure 6A**). The tail currents were larger in irradiated (5 Gy, 2–4 h after irradiation) as compared to control (0 Gy) primary CML cells. This was evident from the peak current densities shown for 0 Gy- and 5 Gy-irradiated cells recorded with -40 mV and +50 mV holding potential (**Figure 6B**) and the thereof calculated holding potential-dependent fraction of peak current densities (**Figure 6C**).

To define the functional significance of radiation-stimulated hERG1-like channel activity, hERG1-dependent cell cycle progression of irradiated (0 or 5 Gy) primary CML cells was assessed by flow cytometry with propidium iodide Nicoletti staining of the DNA 24 h after irradiation (**Figure 6D**). Radiation induced in these cells a decrease of G_1_ and an increase in G_2_ population, suggestive of a G_2_/M arrest (**Figure 6E**, open bars). Importantly, the hERG inhibitor E4031 (3 µM) which was administered during irradiation and the following 24 h post-incubation time blunted the G_2_/M cell cycle arrest (**Figure 6E**, closed bars). This suggests that radiation-induced hERG1 activity contributes to the cellular stress response in primary CML cells similar to its function observed in K562 cells (compare **Figure 4**).

## Discussion

The present study disclosed in the K562 cell line and in primary leukemic cells a function of hERG1 channels for cell cycle control in irradiated CML cells. In K562, radiogenic hERG1 activity contributed to clonogenic survival of the irradiated cells. Activation of K^+^ channels by ionizing radiation has been reported in different tumor entities. In particular, radiation-stimulated activation of Ca^2+^-dependent high conductance BK (K_Ca_1.1, KCNMA1) (Steinle et al., 2011) and intermediate conductance IK (K_Ca_3.1, SK4, KCNN4) K^+^ channels (Stegen et al., 2016) occurs in glioblastoma cells. Radiation stimulated activity of the latter channels has been observed also in human T cell leukemia cells (Klumpp et al., 2016; Voos et al., 2018), in human lung adenocarcinoma (Huber et al., 2012; Roth et al., 2015), and in murine breast cancer cells (Mohr et al., 2019). K_v_3.4 (KCNC4) voltage-gated K^+^ channels (Palme et al., 2013) are activated by ionizing radiation together with hERG1 channels (present study) in K562 cells.

Radiation-induced activation mechanisms may comprise membrane lipid peroxidation and subsequent activation of tyrosine kinases such as src (Dittmann et al., 2009) and Pyk2 kinase (Proudfoot et al., 2018). The latter kinase has been demonstrated to directly modulate BK channels (Stegen et al., 2016). In addition, ionizing radiation has been reported to stabilize hypoxia-inducible factor-1α (HIF1α) which, in turn, upregulates auto-/paracrine chemokine signaling *via* the stromal-cell-derived factor-1 (SDF1, CXCL12)/CXCR4 axis (Edalat et al., 2016). CXCR4 reportedly signals through formation of inositol triphosphate and releases Ca^2+^ from intracellular stores (Hadad et al., 2013), which can be assumed to activate Ca^2+^-dependent K^+^ channels such as IK or BK [for review see (Eckert et al., 2018)]. Beyond that, data in Jurkat T cell leukemia cells suggest that radiogenic activity of IK channels is under the tight control of anti-apoptotic proteins such as Bcl-2 (Klumpp et al., 2016).

hERG channels reportedly contribute to outside-in signaling in complexes with β1 integrin (see *Introduction*) and CXCR4 in the plasma membrane. In addition to radiogenic CXCR4 activation (Pillozzi et al., 2011), ionizing radiation may upregulate integrin β1 signaling by increasing expression and clustering (Voos et al., 2018). One might speculate that radiogenic upregulation of these signaling pathways might underlie the increased hERG1 function in irradiated K562 cells observed in the present study. Combined, this hints to a highly complex regulation mechanisms of Ca^2+^-dependent and voltage-gated K^+^ channel activity in irradiated cells.

Radiogenic K^+^ channel activity has been suggested to drive Na^+^/glucose cotransport across the plasma membrane contributing to glucose fueling of the irradiated cell (Huber et al., 2012). This probably provides extra energy and carbohydrates, the latter required for chromatin remodeling by histone acetylation resulting in DNA decondensation as a prerequisite of DNA damage repair (Dittmann et al., 2013). Moreover, radiogenic K^+^ channel activity reportedly is needed for the repair of radiation-induced DNA double strand breaks (Stegen et al., 2015; Klumpp et al., 2018). Furthermore, radiogenic K^+^ channel activity has been shown to stimulate hypermigration of glioblastoma cells (Steinle et al., 2011; Edalat et al., 2016).

Radiogenic K^+^ channel-mediated electrosignaling reportedly acts both downstream and upstream of Ca^2+^ signaling (Stegen et al., 2016). In theory, Ca^2+^ entry may result in local depolarization of the plasma membrane as well as in local rise in c[Ca^2+^]_free_ beneath the plasma membrane which trigger activation of physically associated voltage-gated K^+^ channels and Ca^2+^-dependent K^+^ channels, respectively. K^+^ channel activity, in turn, maintains the inwardly directed electrochemical driving force for Ca^2+^ entry and/or modulates activity of voltage-regulated Ca^2+^ entry pathway. For instance, in Jurkat T cell leukemia and K562 CML cells, TRPM2 (member 2 of the melastatin family of transient receptor potential) (Klumpp et al., 2016) and TRPV5/6-like (member 5/6 of the vanilloid family of TRP) Ca^2+^-permeable nonselective cation channels (Heise et al., 2010) have been proposed to generate radiogenic Ca^2+^ signals in concert with IK (Klumpp et al., 2016) and K_v_3.4 (Palme et al., 2013) K^+^ channels, respectively. The present study provides evidence that also radiogenic hERG1 activity contributes to those Ca^2+^ signals in K562 cells.

Notably, K_v_3.4 blockage reportedly delays Ca^2+^ re-entry into Ca^2+^-depleted K562 cells (Palme et al., 2013) and decreased in the present study in irradiated cells steady state c[Ca^2+^]_free_ while hERG1 boosted Ca^2+^ re-entry into Ca^2+^-depleted K562 cells and increased c[Ca^2+^]_free_. At the first glance, these opposing effects seem to be counterintuitive since inhibition of both K^+^ channel types is assumed to depolarize the membrane potential of the plasma membrane. One (merely speculative) scenario that might results in opposing K^+^ channel effects in K562 cells assumes that K_v_3.4 channels might be in complex with, e.g., store-operated Ca^2+^ channels and maintain the inwardly directed electrochemical driving force for Ca^2+^ entry by counteracting the Ca^2+^ influx-elicited strong local depolarization of the plasma membrane. hERG1 channels, in contrast might be in complex with further Ca^2+^ entry pathways where they inhibit these pathways either in a non-conductive (i.e., by direct protein-protein interaction) or conductive manner. For the latter, one has to postulate that hERG1 functionally interact with voltage-dependent Ca^2+^ entry pathways that exhibit increasing activity/conductivity with increasing depolarization of the membrane potential.

Likewise, in glioblastoma cells, TRPM8 channels collaborate with Ca^2+^-dependent IK and BK channels (Klumpp et al., 2017) to generate radiogenic Ca^2+^ signals. Similarly to the proposed antagonistic action of K_v_3.4 and hERG1 in K562 cells, IK channel inhibition either decreases steady state c[Ca^2+^]_free_ or induces oscillation in c[Ca^2+^]_free_ in glioblastoma cells while BK channel blockage results in an rapid increase in steady state c[Ca^2+^]_free_ (Stegen et al., 2016). Together, these observations strongly suggest that the radiogenic activities of several K^+^ and Ca^2+^-permeable channel types collaborate to fine tune diverse Ca^2+^ signals in the irradiated cells. Since K_v_3.4 and hERG1 operate during different voltage alterations (K_v_3.4 upon depolarization and hERG1 upon repolarization of the membrane potential) with different kinetics it is hardly surprising that they exert different functions in the Ca^2+^ signalosome.

CaMKII kinases have been identified as downstream targets of radiogenic K^+^ and TRP channel activity [for review see (Stegen et al., 2016)]. Ca^2+^ signals are versatile comprising Ca^2+^ transients, long-lasting Ca^2+^ elevations, oscillations differing in amplitude and frequency, etc. that are formed in a time-spatial dependent manner and that have different meanings for the cells. CaMKII isoforms reportedly are encoded by four genes and expressed in several splice variants that form homo- or hetero-dodecameric CaMKII holoenzymes some of them with nuclear localization signal which exhibit different substrate specificity and an intriguingly complex form of regulation by Ca^2+^/CaM. The latter involves activating and inhibiting autophosphorylation and modulation of CaM binding. Upon stimulatory autophosphorylation at threonine 286 following Ca^2+^/CaM binding, CaMKIIs stay active in the absence of Ca^2+^/CaM, thus, transducing short-term electro- and Ca^2+^ signals in long-lasting biochemical signaling [for review see (Swulius and Waxham, 2008; Coultrap and Bayer, 2012)].

Although resulting in a decrease and increase of c[Ca^2+^]_free_, respectively, inhibition of either hERG1 or K_v_3.4 impaired radiogenic activation of CaMKIIs in K562 cells [(Palme et al., 2013) and present study]. These on the first view conflicting data might reflect a highly complex regulation of the CaMKIIs by Ca^2+^ that require both, K_v_3.4 and hERG1 channels. Nucleus-translocated CaMKII isoforms have shown previously and in the present study to regulate cell cycle in irradiated K562 CML [(Heise et al., 2010) and present study], Jurkat T cell leukemia (Klumpp et al., 2016), and glioblastoma cells (Klumpp et al., 2017). CaMKII-mediated inhibitory phosphorylation of cdc25 phosphatases [cdc25B in K562 CML (Palme et al., 2013) and Jurkat T cell leukemia (Klumpp et al., 2016), and probably cdc25C in glioblastoma cells (Klumpp et al., 2017)] prevents the activating dephosphorylation of the cdc25 target p-(Tyr15)-cdc2 as also observed in the present study. Cdc2 together with cyclin B forms the mitosis-promoting factor, and the inhibitory phosphorylation results in G_2_/M arrest of the cell cycle (Palme et al., 2013; Klumpp et al., 2016; Klumpp et al., 2017).

Importantly, hERG1 as well as K_v_3.4 and IK seem to be critically required not only for CaMKIIs activation but also for cell cycle arrest in irradiated CML [(Palme et al., 2013) and present study), T cell leukemia (Klumpp et al., 2016), and glioblastoma cells (Stegen et al., 2015)], since pharmaceutical or molecular biological targeting of these channels overrides cell cycle arrest resulting in premature (i.e., with unfixed DNA double strand breaks) entry in mitosis and cell death. In addition to cell cycle control, radiogenic hERG1 and K_v_3.4 activities apparently are required to blunt mitochondrial hyperpolarization and superoxide anion formation in irradiated K562 cells (see **Supplementary Figures 2** and **3**). Since mitochondrial Ca^2+^ overload may trigger radiogenic mitochondrial superoxide anion formation [for review see (Eckert et al., 2019)] one might speculate that radiogenic hERG1 and K_v_3.4 activities interfere with Ca^2+^ homeostasis during DNA damage response beyond regulating the Ca^2+^/CaM/CaMKII pathway.

Combined, these observations suggest K^+^ channels such as hERG1 and IK that are upregulated in several tumor entities as a very attractive target for anti-cancer therapy. Preclinical mouse studies have proven the efficacy of an anti-hERG1 therapy strategy (Pillozzi et al., 2011; Crociani et al., 2013; Crociani et al., 2014; Pillozzi et al., 2018). However, since hERG1 contributes to the repolarization of the heart action potential, so-called “torsadogenic” hERG blockers may delay cardiac repolarization and, therefore, substantially increases the risk of Torsade de Pointes and sudden cardiac death. On the other hand, several drugs in clinical use (among those are antihistamines, fluoroquinolone antibiotics, antipsychotics, antiepileptics) exert off-target inhibitory effects on hERG1 partially with submicromolar IC_50_s but are “non-torsadogenic.” One might assume that clinically achieved steady state free plasma concentrations of these “non-torsadogenic” hERG1 inhibitors remain far below its IC_50_ concentration for hERG1 (Lehmann et al., 2018). Another explanation for the “non-torsadogenicity” of these drugs is that hERG1 inhibition alone is not sufficient to induce fatal arrhythmia, and the “torsadogenic” action of a certain drug depends on the full spectrum of ion channels that is modulated by the drug [for commentary see (Arcangeli and Becchetti, 2017)]. Nevertheless, “non-torsadogenic” hERG1 inhibitors might impact tumor biology. Along those lines, a retrospective multivariate analysis of glioblastoma specimens defined hERG1 protein abundance in the tumor as an independent prognostic parameter for shorter patient survival. Notably, patients with highly hERG1-expressing glioblastoma but not those with low expression showed in univariate analysis a benefit in terms of overall survival from “non-torsadogenic” hERG1-inhibiting drugs (Pointer et al., 2017).

Moreover, plasmalemmal hERG1/beta1-integrin complexes seem to be specific for tumor cells (Pillozzi and Arcangeli, 2010) which—at least in theory—increases the possibility to develop tumor-specific inhibitors that target hERG1/beta1-integrin complexes. Finally, the truncated, N-deleted splice variant hERG1b is reportedly up-regulated during the S phase of cell cycle, while the full-length hERG1a protein increases its expression on the plasma membrane during the G1 phase (Crociani et al., 2003). Consequently, proliferating tumor cells should exhibit higher hERG1b/1a expression ratios than terminally differentiated cardiomyocytes (Larsen et al., 2008) as has indeed been reported for some tumors (Erdem et al., 2015). Inhibitor selectivity for variant 1b over 1a (Gasparoli et al., 2015) might be a further strategy to develop tumor-specific hERG1 blockers. Taken together, circumventing cardiotoxic effects of pharmacological hERG1 blockade seems to become feasible in the near future which would classify hERG1 as druggable target in anti-cancer therapy.

In conclusion, irradiated CML cells utilize hERG1-modulated Ca^2+^-signals during DNA damage response. These Ca^2+^ signals contribute *via* the CaMKIIs/cdc2 pathway to cell cycle arrest. Inhibition of hERG1 by the class III antiarrhythmic agent E4031 overrides cell cycle arrest and impairs clonogenic survival of the cells suggesting hERG1 as potential therapy target.

## Data Availability Statement

The datasets generated for this study are available on request to the corresponding author.

## Ethics Statement

The studies involving human participants were reviewed and approved by Ethik-Kommission an der Medizinischen Fakultät der Eberhard-Karls-Universität und am Universitätsklinikum Tübingen. The patients/participants provided their written informed consent to participate in this study.

## Author Contributions

Research design: SH. Conducted experiments: DP, MM, KG, LK. Data analysis: DP, MM, KG, LK, SH. Contributed to discussions: HS, DZ. Wrote or contributed to the writing of the manuscript: HS, DZ, SH. Edited the manuscript: SH. Approved the content and submission of the paper: all authors.

## Funding

This project was supported by a grant of the German Cancer Aid (70112872/70113144). DP was supported by the DFG International Graduate School 1302, ). LK was supported by the ICEPHA program of the University of Tübingen and the Robert-Bosch-Gesellschaft für Medizinische Forschung, Stuttgart, Germany.

## Conflict of Interest

The authors declare that the research was conducted in the absence of any commercial or financial relationships that could be construed as a potential conflict of interest.
