# Supplementary material for: hERG K+ Channels Promote Survival of Irradiated Leukemia Cells
Source: Front Pharmacol. 2020 Apr 24;11:489. doi: 10.3389/fphar.2020.00489 (PMC7194033; doi:10.3389/fphar.2020.00489)
Supplement: Supplementary file 1 [file DataSheet_1.pdf]

Supplementary Material

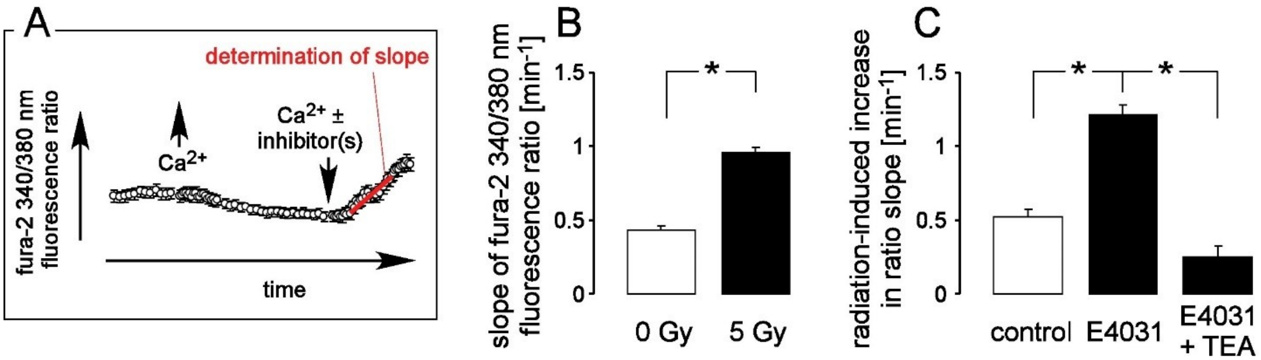

Suppl. Fig. 1. hERG1 blockage by E4031 augments radiation-induced Ca^{2+} re-entry in Ca^{2+}-depleted K562 cells, an effect abolished by additional TEA-mediated inhibition of $K_v3.4$ channels. **A.** Scheme illustrating the Ca^{2+} removal/re-addition protocol applied in fura-2 Ca^{2+} imaging experiments (see main article). $c[Ca^{2+}]_{free}$ was determined 1-4 h post-irradiation with 0 or 5 Gy during superfusion with Ca^{2+}-containing NaCl solution (see main article), during a 5 min-period of extracellular Ca^{2+} removal in EGTA-buffered NaCl solution (in mM: 125 NaCl, 32 HEPES, 5 KCl, 5 D-glucose, 1 $MgCl_2$, 0.5 EGTA, titrated with NaOH to pH 7.4), and during Ca^{2+} re-addition in Ca^{2+}-containing NaCl solution additionally containing vehicle, E4031 or E4031 plus isosmotically added TEA (see main article). During Ca^{2+} re-addition, the slope of the increase in fura-2 340/380 nm fluorescence ratio was determined for the linear course of ratio increase (indicated by red line) as a measure of Ca^{2+} re-entry. **B, C.** Mean (± SE, n = 167-267) slope of fura-2 ratio increase upon Ca^{2+} re-addition in control and irradiated K562 cells (B) and (C) radiation-induced increase in fura-2 ratio slope recorded in the absence (B and C, 1st bar) and presence of E4031 (C, 2nd bar) or E4031 and TEA (C, 3rd bar). * indicates $p \le 0.05$, ANOVA.

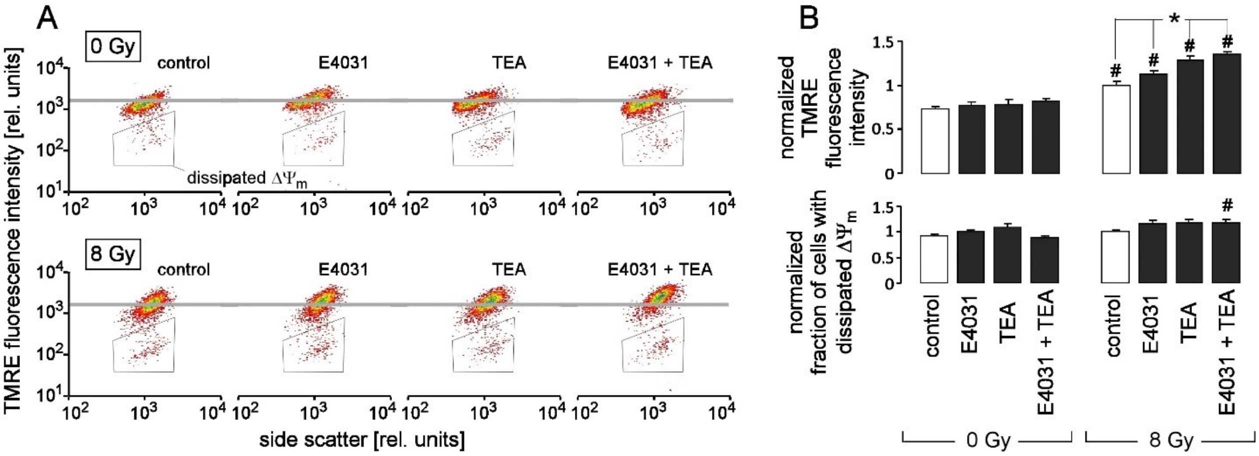

Suppl. Fig. 2. $K_v3.4$ channel blockage and in tendency also hERG1 inhibition induce hyperpolarization of the inner mitochondrial membrane potential ($\Delta\Psi_m$) in irradiated K562 cells. **A.** Dot plots recorded by flow cytometry showing the $\Delta\Psi_m$-specific TMRE fluorescence and the side scatter of K562 cells 24 h after irradiation with 0 Gy (upper line) or 8 Gy (lower line). K562 cells were irradiated (0 or 8 Gy) and further incubated for 24 h in the presence of E4031 (0 and 3 µM) or TEA (0 or 3 mM) or both inhibitors as indicated. Grey lines are introduced to better visualize the changes in TMRE fluorescence between the experimental conditions. The gates define the cells with dissipated $\Delta\Psi_m$. **B.** Mean (± SE, n = 10-12) TMRE fluorescence intensity of the cells with non-dissipated $\Delta\Psi_m$ (upper bar diagram) and percentage of cells with dissipated $\Delta\Psi_m$ (lower bar diagram) as a function of irradiation and hERG1 and/or $K_v3.4$ inhibition. The data were normalized to those of the irradiated controls (8 Gy without inhibitors). $^{\#}$ and * indicate $p \leq 0.05$, ANOVA, $^{\#}$ describes the difference between the irradiated and the respective unirradiated situation.

K562 cells were irradiated and 24 h post-incubated in the absence and presence of the inhibitors in cell culture medium. To control for the increase in osmolarity or any dilution effects by addition of TEA (3 mM), NaCl (3 mM) was added to all cell culture media not containing TEA. Harvested cells were incubated with TMRE (tetramethylrhodamine ethyl ester perchlorate, 25 nM final concentration, Invitrogen, Thermo Fisher Scientific) in cell culture medium for 30 min at 37°C and TMRE fluorescence was recorded by flow cytometry in fluorescence channel FL-2 (564-606 nm emission wavelength, logarithmic scale) and analyzed as geometrical means (cells with non-dissipated $\Delta\Psi_m$) and percentage of gated cells (cells with dissipated $\Delta\Psi_m$).

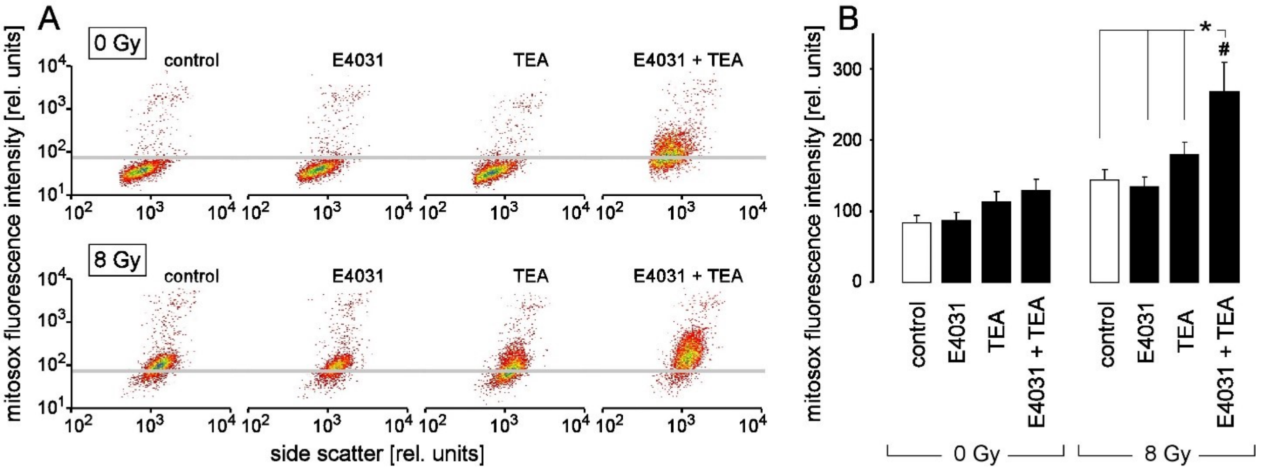

Suppl. Fig. 3. Combined blockage of hERG1 and K_v3.4 channels promotes mitochondrial superoxide anion formation in irradiated K562 cells. **A.** Dot plots recorded by flow cytometry showing the superoxide anion-specific mitosox fluorescence and the side scatter of K562 cells 24 h after irradiation with 0 Gy (upper line) or 8 Gy (lower line). K562 cells were irradiated (0 or 8 Gy) and further incubated for 24 h in the presence of E4031 (0 and 3 µM) or TEA (0 or 3 mM) or both inhibitors as indicated. Grey lines are introduced to better visualize the changes in mitosox fluorescence between the experimental conditions. **B.** Mean (± SE, n = 12) mitosox fluorescence intensity as a function of irradiation and hERG1 and/or K_v3.4 inhibition. # and * indicate $p \leq 0.05$, ANOVA, # describes the difference between the irradiated and the respective unirradiated situation.

K562 cells were irradiated and 24 h post-incubated in the absence and presence of the inhibitors in cell culture medium. To control for the increase in osmolarity or any dilution effects by addition of TEA (3 mM), NaCl (3 mM) was added to all cell culture media not containing TEA. Harvested cells were incubated for exact 20 min (mitosox fluorescence increase with dye incubation time) at 37°C with mitosox (5 µM, Invitrogen, Thermo Fisher Scientific). Mitosox fluorescence was recorded by flow cytometry in fluorescence channel FL-2 (564-606 nm emission wavelength, logarithmic scale) and analyzed as geometrical means. To confirm equal fluorescence dye loading, recorded samples were oxidized (10 mM *tert*-butylhydroperoxide) for 10 min and re-recorded (data not shown). # and * indicate $p \leq 0.05$, ANOVA, # describes the difference between the irradiated and the respective unirradiated situation.
